# Label-free relative quantitative proteomics reveals extracellular vesicles as a vehicle for *Salmonella* effector protein delivery

**DOI:** 10.3389/fmicb.2022.1042111

**Published:** 2022-12-15

**Authors:** Tao Wu, Biao Zhang, Juane Lu, Ailin Huang, Hao Wu, Jianjun Qiao, Haihua Ruan

**Affiliations:** ^1^Tianjin Key Laboratory of Food Science and Biotechnology, College of Biotechnology and Food Science, Tianjin University of Commerce, Tianjin, China; ^2^Key Laboratory of Systems Bioengineering, Ministry of Education, Department of Pharmaceutical Engineering, School of Chemical Engineering and Technology, Tianjin University, Tianjin, China

**Keywords:** extracellular vesicles, comparative proteomics, *Salmonella enterica* serovar typhimurium, effector protein, SopB

## Abstract

Extracellular vesicles are small vesicles with a diameter of 30–150 nm that are actively secreted by eukaryotic cells and play important roles in intercellular communication, immune responses, and tumorigenesis. Previous studies have shown that extracellular vesicles are involved in the process of *Salmonella enterica* serovar Typhimurium (*S.* Typhimurium) infection. However, changes in the protein content of extracellular vesicles elicited by *S.* Typhimurium infection have not been determined. Here, we extracted the extracellular vesicles with high purity from *S.* Typhimurium-infected Henle-407 cells, a kind of human intestinal epithelial cells, by ultracentrifugation combined with an extracellular vesicles purification kit, and analyzed their protein composition using label-free relative quantitative proteomics. The extracted extracellular vesicles exhibited an oval vesicular structure under electron microscopy, with a mean diameter of 140.4 ± 32.4 nm. The exosomal marker proteins CD9, CD63, and HSP70 were specifically detected. Compared with the uninfected group, nearly 1,234 specifically loaded proteins were uncovered in *S.* Typhimurium-infected Henle-407 cells. Among them were 409 *S*. Typhimurium*-*derived specific proteins, indicating a significant alteration in protein composition of extracellular vesicles by *S.* Typhimurium infection. Notably, these proteins included 75 secretory proteins and over 300 non-secretory proteins of *S.* Typhimurium, implicating novel pathways for bacterial protein delivery, although it remains unclear if their loading into extracellular vesicles is active or passive. To investigate the roles of these extracellular proteins, we exemplified the function of SopB, a well-known T3SS effector protein, and showed that the extracellular SopB could be taken up by RAW264.7 macrophages, activating the phosphorylation of Akt. This study provides new insights into the mechanism of *Salmonella* infection through extracellular vesicles that transport virulence proteins to uninfected neighboring cells to facilitate further infection.

**Figure:**
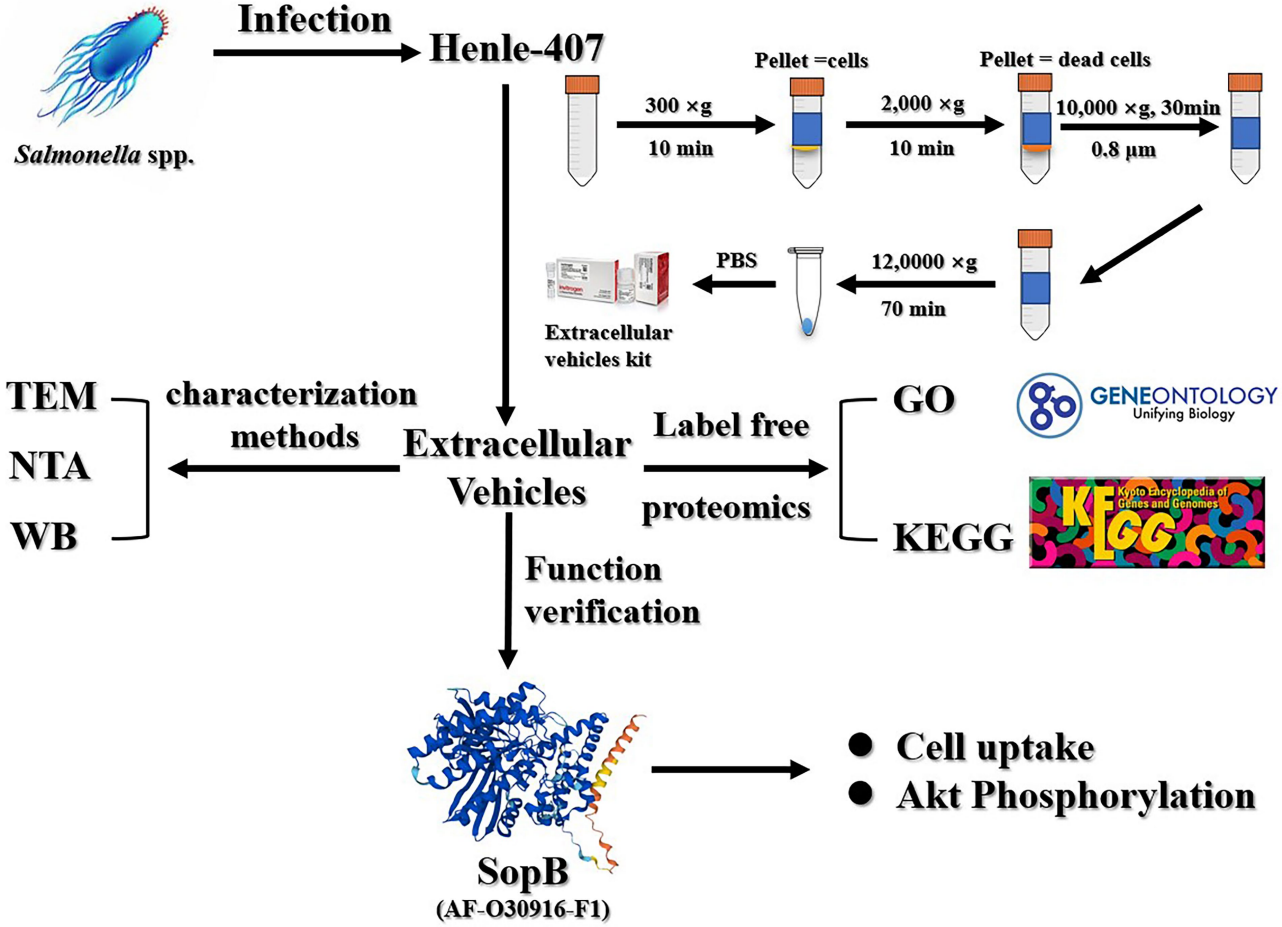
Graphical Abstract

## Introduction

*Salmonella enterica* serovar Typhimurium (*S.* Typhimurium) has become a cause for major public health concern worldwide and emerged as an important food-borne pathogen resulting in a considerable public health burden (Majowicz et al., 2010; Ao et al., 2015). *S.* Typhimurium invades the gastrointestinal tract through oral ingestion of contaminated food or water (Pocsfalvi et al., 2016). *Salmonella* secretes proteins through specific secretion systems and these secreted proteins act as virulence factors (also known as effector proteins) to invade and regulate various functions of host cells, including inhibiting host immune responses (Qaidi et al., 2018), promoting the survival of intracellular bacteria (Haraga et al., 2008), and plasma membrane transport of target products (Santos et al., 2020).

*Salmonella* effector proteins mainly enter the host cells through three pathways, including a type III secretion system (T3SS; Santos et al., 2020), type IV secretion system (T4SS; Gokulan et al., 2013) and type VI secretion system (T6SS; Silverman et al., 2012; Brunet et al., 2014; Sana et al., 2016; Sibinelli-Sousa et al., 2020). Studies indicate that the supramolecular structure of T3SS resembles a needle, which is now commonly referred to as the T3SS injection structure. This structure directly delivers *S*. Typhimurium effector proteins into the host cell and aids the bacterial colonization (Notti and Stebbins, 2016; Santos et al., 2020). Different from the T3SS, the T4SS not only transports effector proteins, but also delivers DNA into the host cells (Galán and Waksman, 2018), which is mainly involved in regulating the adaptation of *Salmonella* to the macrophage intracellular environment and responsible for encoding proteins involved in intestinal mucosal fluid secretion and inflammatory response (Jesús et al., 2018). Distinctly, T6SS is a dynamic organelle that bacteria use to target prey cells for inhibition *via* translocation of effector proteins (Cascales and Cambillau, 2012), which inhibit host Th1 and Th2 immune responses to facilitate persistent colonization of the host by *S. enterica* serovar Pullorum (Hxa et al., 2020). However, all three pathways require direct contact of the bacteria with host cells for effector proteins to enter the cytoplasm.

Extracellular vesicles (EVs) are lipid bound vesicles secreted by cells into the extracellular space. The three main subtypes of EVs are microvesicles, exosomes, and apoptotic bodies (Doyle and Wang, 2019). Exosomes are small membrane-bound vesicles with diameters of 30 to 150 nm that are released by many different cell types, although they were considered to be only a mechanism of transferrin removal from mature reticulocytes (Pan et al., 1985; Sook et al., 2014). The secretory proteome of human J774 macrophages infected with *M. tuberculosis* demonstrated that some of the proteins are released *via* exosomes. LC–MS/MS proteomic analysis of exosomes released by macrophages infected with *M. tuberculosis* identified a total of 41 mycobacterial proteins, as well as some tuberculosis-specific host membrane proteins and lipoproteins, which stimulate the activation of dendritic cells, promote their antigen presentation to T cells, and elicit acquired immune responses (Athman et al., 2017). To our knowledge, these pro-inflammatory exosomes could be formed in the early phase of macrophage infection by *S.* Typhimurium, and were through to transfer cargo proteins of pathogenic bacteria to neighboring non-infected cells, thereby leading to their activation by pathogens (Hui et al., 2018).

Subsequently, it was hypothesized that extracellular vesicles might be a previously overlooked way for invasive pathogens to transport their effector proteins. They are initially generated by inward budding of late endosomes, which results in the formation of multivesicular bodies (MVBs) in the cytosol. These MVBs are subsequently transported to the extracellular space by fusion with the plasma membrane and exocytosis (Daniel et al., 2015). Extracellular vesicles contain many types of bioactive components such as proteins, lipids, and nucleic acids, among others. They participate in immuno-stimulation and protein secretion (Niel et al., 2018). In general, extracellular vesicles act as carriers of intercellular signal molecules (Tkach and Théry, 2016).

Here, we systematically analyzed the extracellular vesicles secreted by host cells after *S.* Typhimurium infection through label-free relative quantitative proteomics and found that they contain 409 *Salmonella* proteins, including 75 secretory proteins and over 300 non-secretory proteins of *S.* Typhimurium. Here, we exemplified the role of the *Salmonella* effector protein SopB, a well-known effector protein that plays important roles in protecting host cells from apoptosis by inducing sustained Akt activation (Cooper et al., 2011), which can be transported into macrophages *via* extracellular vesicles from intestinal epithelial cells infected with *S*. Typhimurium, and cause the phosphorylation of Akt protein. These findings suggest that extracellular vesicles secreted by host cells may serve as a previously overlooked pathway for the transport of virulence proteins to other recipient cells during infection.

## Materials and methods

### Bacterial strains, cells, growth conditions, and reagents

Wild-type (WT) *Salmonella enterica* serovar Typhimurium LT2, described by Li et al. (2007), was used in this study. The Δ*sopB* mutant strain was constructed using the λ-Red recombination system (Datsenko and Wanner, 2000). The Henle-407 human intestinal epithelial cells and RAW 264.7 macrophages were purchased from the American Type Culture Collection (ATCC). All cells were cultured in antibiotic-free Dulbecco’s modified Eagle medium (DMEM; Gibco) supplemented with 10% fetal bovine serum (FBS) at 37°C under a 5% CO_2_ atmosphere.

Rabbit anti-phospho-Akt and rabbit anti-Akt antibodies were purchased from Cell Signaling Technology (Beverly, CA, United States). Rabbit anti-CD9, anti-CD63, anti-HSP70 and a secondary anti-rabbit antibody were purchased from System Biosciences (Bay Area, CA, United States). The ExoRNeasy Serum/Plasma Maxi Kit was purchased from Qiagen (Valencia, CA, United States).

### Bacterial infection

The Henle-407 human intestinal epithelial cells were cultured in high sugar DMEM medium containing 10% fetal bovine serum (FBS) and the cells were incubated in an incubator at 37°C with 5% CO_2_ saturated humidity and digested with 0.25% trypsin for passaging. Cells at 80% confluence were starved in FBS-deficient DMEM for 2 h. *S.* Typhimurium was cultured in liquid LB medium after activation until logarithmic growth and the organisms were collected during this period. Then, the starved cells were infected with *S.* Typhimurium at a multiplicity of infection (MOI) of 30 for 30 min. The infected cells were subsequently incubated with DMEM containing 100 μg/ml gentamicin for 30 min. Finally, the infected cells were washed twice with pre-warmed phosphate-buffered saline (PBS) and then incubated with DMEM containing 10 μg/ml gentamicin for 8 h until harvesting cell culture media supernatant (Ruan et al., 2017).

### Extracellular vesicles isolation, transmission electron microscopy, nanoparticle tracking and Western blot analysis

In order to acquire high purity extracellular vesicles, extracellular vesicles were prepared by classical ultracentrifugation combined with the exoRNeasy Serum/Plasma Maxi kit. Briefly, 15 dishes (10 × 10 cm) of cell cultures after infection or without infection were incubated for the indicated times, collected and filtered through a 0.8 μm pore-size filter. Then, the 150 ml of collected cell culture supernatants were subject to differential centrifugation at 300 × *g* for 10 min, 2000 × *g* for 10 min, and 10,000 × *g* for 60 min, respectively. Next, the supernatant was subject to ultracentrifugation at 120,000 × *g* for 70 min, and the sediment was resuspended to a volume of 10 ml in PBS. Finally, the extracellular vesicles were further prepared by extraction according to the exoRNeasy Maxi Kit’s instructions.

To visualize extracellular vesicles *via* transmission electron microscopy (TEM), the extracellular vesicle pellets were placed in a droplet containing 2.5% glutaraldehyde in PBS buffer at pH 7.2 and fixed overnight at 4°C for conventional TEM. The samples were rinsed in PBS buffer (3 times, 10 min each) and then fixed in 1% osmium tetroxide for 60 min at room temperature. The samples were embedded in 10% gelatin, fixed in glutaraldehyde at 4°C, and cut into several blocks (smaller than 1 mm^3^). The samples were dehydrated for 10 min per step in increasing concentrations of ethanol (30, 50, 70, 90, 95, and 100%, three times each). Next, the pure ethanol was exchanged with propylene oxide, and the specimens were infiltrated with increasing concentrations (25, 50, 75, and 100%) of Quetol 812 epoxy mixed with propylene oxide for a minimum of 3 h per step. The samples were embedded in pure, fresh Quetol 812 epoxy and polymerized at 35°C for 12 h, 45°C for 12 h, and 60°C for 24 h. Ultrathin sections (100 nm) were cut using a Leica UC6 ultramicrotome and post-stained with uranyl acetate for 10 min and lead citrate for 5 min at room temperature, prior to observation using a FEI Hitachi HT-7700 transmission electron microscope operated at 120 kV.

The size of the extracellular vesicle was measured by suspending exosomes in PBS buffer prior to analysis on a Zeta VIEW nanoparticle tracking analyzer (Particle Metrix, Germany).

Extracellular protein detection was performed by western blot analysis using a previously described chemiluminescence method (Ruan et al., 2014). A 50 μl sample of extracellular vehicles was prepared, transferred to a membrane after SDS-PAGE electrophoresis, and closed with 5% nonfat milk. The primary antibody was diluted 1:1000 by volume and incubated overnight at 4°C. The samples were washed 3 times with TBST solution and secondary antibody diluted 1:2,000, incubated for 2 h at room temperature, washed 3 times with TBST solution, then ECL was added for color development, and imaged using Tanon 5,200 Automatic Chemiluminescence Image Analysis System. The abundance of protein bands in images acquired from western blots was quantified using Image J software.

### Label-free relative quantitative proteomic analysis of the extracellular vesicles

Quantitative proteomic analysis of the extracellular vesicles mainly included protein extraction, enzymatic hydrolysis to generate peptides, and HPLC–MS/MS Data collection.

Extracellular proteins were extracted by SDT (4% (W/V) SDS, 100 mM Tris/HCl pH 7.6, 0.1 mol/l DTT), and then quantified using the BCA method. An appropriate amount of protein was taken from each sample for trypsin enzymolysis using the Filter Aided Proteome preparation (FASP) method (Wiśniewski et al., 2009). A C18 cartridge was used to desalt the peptides, which were then lyophilized and re-dissolved in 40 μl 0.1% formic acid solution for peptide quantification.

For LC–MS/MS data collection, each sample was first separated by HPLC on an Easy-nLC system at a nanoliter flow rate. Buffer A was 0.1% formic acid aqueous solution, and buffer B was 0.1% formic acid in 84% acetonitrile aqueous solution. The chromatographic column was balanced with 95% liquid A, and the samples were loaded to the loading column (Thermo Scientific Acclaim PepMap100, 100 μm × 2 cm, nano Viper C18) by the automatic sampler. The separation was performed on an analytical column (Thermo Scientific EASY Column 10 cm, ID75 μm, 3 μm, C18-A2) at a flow rate of 300 nl/min.

After chromatographic separation, the samples were analyzed using a Q-Exactive mass spectrometer (Thermo Fisher) in positive ion mode. The scanning range of parent ions was 300–1800 m/z, the resolution of first-level mass spectrometry was 70,000 at 200 m/z, the Automatic Gain control (AGC) target was 1e^6^, and the Maximum IT was 50 ms. The dynamic exclusion time was 60.0 s. The mass charge ratios of peptides and peptide fragments were collected as follows: 20 fragment profiles (MS2 Scan) were collected after each full scan; MS2 activation type was HCD, and the isolation window was 2 m/z. The secondary mass spectrometry resolution was 17,500 at 200 m/z. Normalized collision energy was 30 eV, and underfill was 0.1%.

### Identification and quantitation of proteins

The MS raw data for each sample were searched using the MaxQuant (Max Planck Institute of Biochemistry, Munich, Germany, version 1.5.3.17) for identification and quantitation analysis. The protein FDR ≤ 0.01, peptide FDR ≤ 0.01, MS/MS Tolerance was 20 ppm. Database used in the research was uniport_ *Salmonella_enterica*_serovar_Typhimuriun_99287. LFQ/iBAQ was used for Protein Quantification. Related parameters and instructions are in details as Table 1 shown.

**Table 1:** Parameters and instructions for identification and quantitation of proteins.

**Item**	**Value**
Enzyme	Trypsin
Max Missed Cleavages	2
Fixed modifications	Carbamidomethyl (C)
Variable modifications	Oxidation (M)
Main search	6 ppm
First search	20 ppm
MS/MS Tolerance	20 ppm
Database	uniprot_*Salmonella*_enterica_serovar
	_Typhimurium_99287.fasta
Database pattern	Reverse
protein FDR	≤0.01
Peptide FDR	≤0.01
Protein Quantification	LFQ/iBAQ

### Bioinformatics analysis

Gene ontology (GO) term annotations of differentially abundant proteins in extracellular vesicles isolated from Henle-407 cells after *S.* Typhimurium infection compared to uninfected cells was performed using Blast2GO (version 2.6.4)^1^ with the default annotation parameters (BLAST e-value threshold of 1e-06, Gene Ontology annotation threshold of 55). Kyoto Encyclopedia of Genes and Genomes (KEGG) pathway annotation was performed using the KEGG Automatic Annotation Server (KAAS) with the bi-directional best-hit method. Functional enrichment analysis for statistical significance of GO terms and KEGG pathways between the differential protein sets in extracellular vesicles isolated from Henle-407 cells after *S.* Typhimurium infection compared to uninfected cells was performed using Fisher’s exact test with default parameters.

### Extracellular vesicles uptake assay

Firstly, an appropriate amount of was collected and re-suspended in 500 μl BSA (1,000 μg/ml), which was named tube A. At the same time, 500 μl BSA (1,000 μg/ml) and 4 μl PKH67 were added to a sterile 1.5 ml tube, and fully mixed to form tube B. Next, tubes A and B were mixed and incubated at room temperature for 5 min, after which 1% BSA in PBS was added to stop dyeing for 1 min, and the volume of the mixture was increased to 20 ml by adding 1% BSA. After that, the sample was centrifuged at 120,000 g for 60 min and the supernatant was carefully discarded, after which the precipitate was dissolved in sterile PBS and named PKH67-EVs. Secondly, RAW264.7 cells in good condition were seeded onto a Millicell EZ Slide (5,000 cells/well) and cultured overnight with 500 μl medium, after which the RAW264.7 cell culture medium was removed and cells were rinsed with pre-warmed DMEM 3 times, after which 200 μl DMEM was added. The freshly prepared PKH67-EVs was collected, added to Raw264.7 cells and incubated at 37°C with 5% CO_2_ for 4 and 8 h. After incubation, the culture medium was discarded and the cells were rinsed with PBS 3 times. The cells were then fixed with 4% paraformaldehyde for 10 min. After the paraformaldehyde was removed, the cells were rinsed with sterile PBS 3 times, the slides were stained with DAPI and fixed with resin. Finally, the uptake of extracellular vesicles was observed by confocal microscopy.

### Statistical analysis

Unless otherwise noted, the results were presented as the means ± standard deviations for three independent replicates. Statistical significance was calculated using one-tailed paired Student *t*-test or two-way analysis of variance (ANOVA) in Prism software (GraphPad Software, Inc., United States). *p* values of <0.05 were considered to indicate significant differences.

## Results

### Characterization of the extracellular vesicles isolated from *S.* typhimurium-infected human intestinal epithelial Henle-407 cells

**Figure 1:**
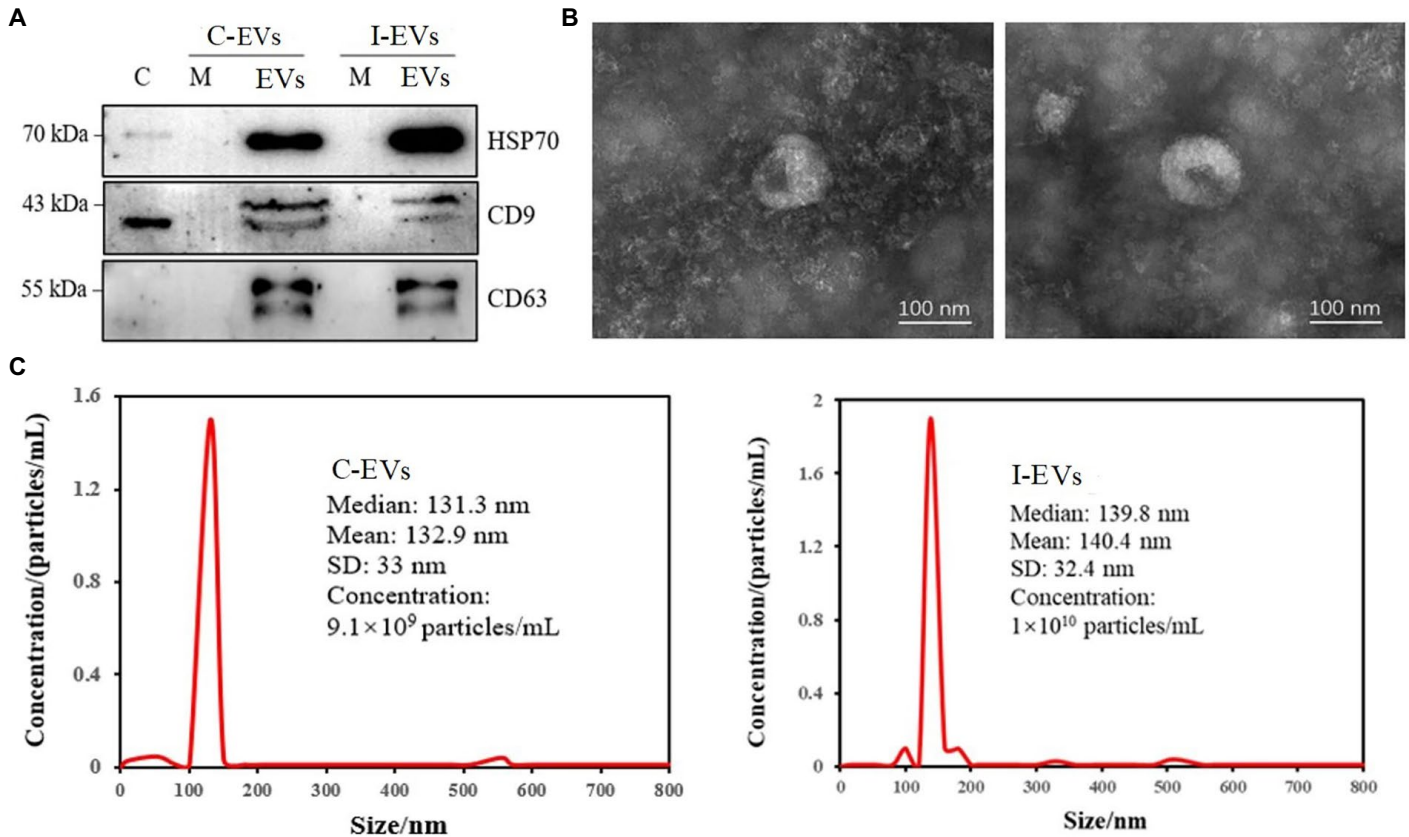
Characterization of the isolated extracellular vesicles. C-EVs denotes the extracellular vesicles produced by uninfected Henle-407 cells. I-EVs denotes the extracellular vesicles produced by Henle-407 cells infected with *S.* Typhimurium at 8 hpi. **(A)** Western blot analysis showing the specific presence of HSP70, CD63, and CD9 in the extracellular vesicles. C denotes the whole cell lysate, M denotes the cell culture medium, and EVs denotes the extracellular vesicles of Henle-407 cells. **(B)** Extracellular vesicles obtained from uninfected (left) and *S.* Typhimurium-infected (right) Henle-407 cells were visualized by transmission electron microscopy. **(C)** Size distribution of the obtained extracellular vesicles derived from uninfected (left) and *S.* Typhimurium-infected (right) Henle-407 cells. The mean size of the extracellular vesicles (nm) and their concentrations (number of particles/mL) were recorded using a nanoparticle tracking instrument.

To confirm the quality of extracellular vesicles obtained through ultracentrifugation combined with a commercial purification kit, the exosome-marker proteins HSP70, CD9 and CD63 (Théry et al., 2018) were validated by western blot analysis. As shown in Figure 1A, there was low or non-detectable presence of marker proteins in the whole cell lysate (C) and cell culture medium supernatant (M). By contrast, all three specific markers were successfully detected in the extracellular vesicles isolated from both *S.* Typhimurium-infected (I-EVs) and uninfected Henle-407 cells (C-EVs), showing that the extracellular vesicles were successfully isolated.

For further characterization, the isolated extracellular vesicles were subjected to transmission electron microscopy (TEM). The images showed homogenous membranous vesicles ranging from 30 to 150 nm in diameter, with a characteristic spherical or oval appearance (Figure 1B), which was consistent with the previous results (Sook et al., 2014). There were no significant differences in the morphology/size of extracellular vesicles prepared from *S.* Typhimurium-infected and uninfected Henle-407 cells (Figure 1B). Detection for tracking the size distribution and diameter of nanoparticles indicated that the peak diameter of extracellular vesicles from the C-EVs group was 132.9 ± 33 nm, and the diameter of extracellular vesicles from the I-EVs group was 140.4 ± 32.4 nm (Figure 1C), which confirmed the successful isolation and purification of extracellular vesicles suitable for subsequent quantitative proteomic analysis.

### Label-free quantitative proteomic analysis of the extracellular vesicles revealed the presence of *S.* typhimurium virulence proteins

**Figure 2:**
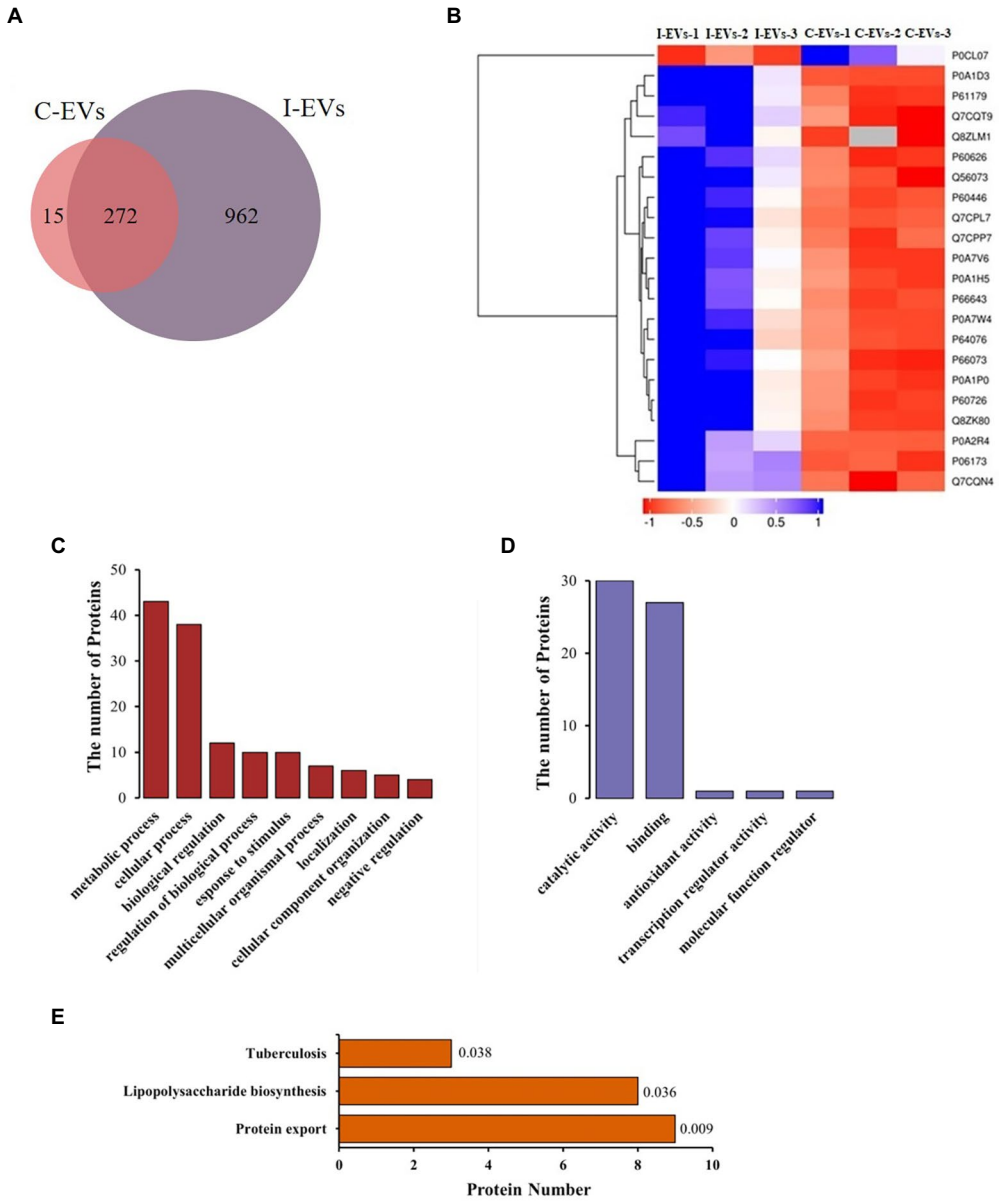
Label-free quantitative proteomics analysis of the isolated extracellular vesicles from uninfected or *S.* Typhimurium-infected Henle-407 cells at 8 hpi. **(A)** Venn diagram showing the detected proteins in two groups. **(B)** Heatmap illustrating the upregulated proteins among the overlapping proteins between the two groups. Additional information is available in Supplementary Table S1. **(C,D)** GO analysis showing the total protein counts stratified by biological process and molecular function. The *S.* Typhimurium protein database was used as a reference protein list for the fold enrichment analysis and a Bonferroni correction for multiple testing was used in each case. The top GO terms were chosen based on the statistical significance (the smallest *p* value), and the fold enrichment for each term is shown. **(E)** Enrichment map of KEGG pathways of the differentially abundant proteins.

The extracellular vesicles isolated from *S.* Typhimurium-infected and uninfected Henle-407 cells were analyzed by label-free quantitative proteomics. In this study, we simultaneously analyzed human and bacterial proteins within extracellular vesicles, focusing on the composition and function of the extracellular bacterial proteins. As shown in Supplementary Table S1, a total of 1,280 proteins were identified in the *S.* Typhimurium infected and control group when searching the mass spectra of the identified peptides against the *S*. Typhimurium LT2 database. After excluding the 31 proteins that appeared only once in each of the three parallel repeats in each group, we found that 272 (22% of the total) proteins were found in the extracellular vesicles from both the *S.* Typhimurium infected and uninfected groups as shown in Figure 2A. These 272 overlapping proteins were not *S.* Typhimurium derived proteins due to their presence in the uninfected control group. In fact, they were human proteins that exist within extracellular vesicles but were unavoidably detected by peptide fraction searches in the *S.* Typhimurium LT2 protein database due to their high conservation between *Salmonella* and human cells, they were simultaneously detected in both groups (Figure 2A). These proteins included structural proteins, ribosomal proteins, dihydrolipoyl dehydrogenase, and outer membrane porins. Among the overlapping proteins, differentially expressed proteins were screened with multiple changes of more than 2.0 times (upregulated by more than 2-fold or down-regulated to less than 0.5 fold) and *p* < 0.05. There were 22 differentially expressed proteins, including 21 up-regulated proteins and 1 down-regulated protein in extracellular vesicles between the infected and uninfected groups (Figure 2B).

In order to better describe the role of the specific *S*. Typhimurium proteins present in the extracellular vesicles secreted by Henle-407 cells during infection, we obtained 409 *S*. Typhimurium proteins (Supplementary Table S2) from 962 proteins according to a screening standard that the proteins were not expressed in the three replicates of the control group but at least simultaneously identified in two of three replicates from the *S.* Typhimurium infected group.

Subsequently, Blast2go (Stefan et al., 2008) was used for Gene Ontology (GO) analysis of these 409 proteins, and Fisher’s exact test was used to screen significantly enriched proteins. The traditional GO analysis is based on enrichment analysis in the three categories of cellular component, biological process and molecular function. As the source of these 409 proteins was *S.* Typhimurium, we only analyzed the biological process and molecular function of these *S.* Typhimurium specific proteins. As shown in Figure 2C, the biological process annotations uncovered that these 409 proteins were mainly involved in metabolic processes, cellular processes, biological regulation, regulation of biological processes, response to stimuli, multicellular organismal processes, localization, cellular component organization and negative regulation. The total counts of proteins in each of these biological processes were 43, 38, 12, 10, 10, 7, 6, 5, and 4, respectively. The molecular function diagrams of the above proteins were also drawn based on GO analysis (Figure 2D), including catalytic activity, binding activity, antioxidant activity, transcriptional regulatory activity and molecular function regulatory activity. The total counts of proteins in each functional classification were 30, 27, 1, 1, and 1, respectively. GO analysis showed that these 409 *S.* Typhimurium proteins were mainly involved in metabolic and cellular processes, mostly exerting molecular functions such as catalysis and binding activities.

Similarly, Kyoto Encyclopedia of Genes and Genomes (KEGG) enrichment analysis of 409 *S.* Typhimurium proteins within the extracellular vesicles was also carried out. As shown in Figure 2E, there were 20 proteins associated with 3 KEGG pathways, including protein export, lipopolysaccharide biosynthesis and tuberculosis. The total counts of specific *S*. Typhimurium proteins involved in the three pathways were 9, 8 and 3, while the corresponding *p* values were 0.009, 0.036 and 0.038, respectively. Our results showed that the protein secretion pathway was significantly different from other two pathways and was the most enriched among the specific *Salmonella* proteins. However, the prediction and analysis of the functions of these extracellular *S.* Typhimurium proteins in regulating adjacent cells or long-distance host cells has been technically limited until now.

### Prediction of secretion and functional analysis of extracellular *S*. typhimurium proteins

**Figure 3:**
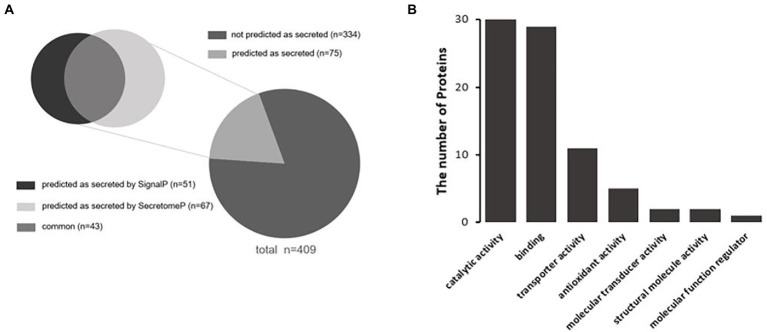
**(A)** Prediction of secreted proteins among *Salmonella* proteins with significant changes in abundance within extracellular vesicles. SecretomeP was used for *ab-initio* predictions of non-classical (i.e., not signal peptide mediated) protein secretion, and SignalP was used for predictions of secreted proteins with N-terminal signal peptides. **(B)** Functional classification of secretory proteins by GO analysis.

Extracellular Vesicles are important carriers of signals for intercellular communication, and all their content is derived from the source cells, including the bacterial proteins identified in this study. However, pathways that might be employed by the 409 *S*. Typhimurium proteins to enter the host cells that produced the extracellular vehicles were unclear. In this study, Signal-5.0 and SecretomeP 2.0 software was used to predict the possible secretion signals of these 409 *Salmonella* proteins. It was observed that more than 18% of the proteins in this subset were predicted to be secreted from the bacterial cell (Figure 3A). An N-terminal signal peptide was found in 16.4% of the proteins, which included Trk system potassium uptake protein TrkA, tail-specific protease Prc, thioredoxin reductase TrxB, and the chaperone SurA (Supplementary Table S3). There were also 12.5% predicted targets of non-canonical secretion according to SecretomeP 2.0 (Bendtsen et al., 2004), which included the 30S ribosomal protein S14 RpsN, inositol phosphate phosphatase SopB, RNA-binding protein Hfq, and cold shock-like protein CspC (Supplementary Table S4). However, the remaining 334 proteins were not predicted as secreted (Figure 3A; Supplementary Table S2), including acetyl-coenzyme A carboxylase carboxyl transferase subunit alpha accA, esterase FrsA, catabolite repressor/activator Cra, and UDP-3-O-acyl-N-acetylglucosamine deacetylase lpxC. Subsequently, the molecular functions of 75 secretory proteins were classified based on GO analysis to clarify their effects on the regulation of host cell physiology. It was demonstrated that the molecular functions of secretory proteins were mainly related to catalytic activity, binding, transporter activity, antioxidant activity, molecular transducer activity, structural roles and regulation of molecular function (Figure 3B). The total counts of the *S.* Typhimurium proteins in each molecular functional classification were 30, 29, 11, 5, 2, 2, and 1, respectively. The molecular function of these secretory proteins was basically the same as that of the 409 proteins in terms of classification and quantity (Figure 2D). Thus, we concluded that the secretory proteins within extracellular vesicles produced by *S.* Typhimurium infected host cells play important roles in regulating the physiological functions of adjacent cells or long-distance host cells, implicating possible roles during bacterial infection.

Finally, we analyzed the functions of these 75 secretory proteins to reveal the main aspects of their role in the extracellular vesicles. Ten typical virulence proteins related to the infectivity of *S.* Typhimurium (Table 2) were identified, 7 of which were related to outer membrane assembly of Gram-negative bacteria and 3 to T3SS effector proteins of *S.* Typhimurium, including SopB, SipD and SctC. It is known that *S.* Typhimurium mainly secrets effector proteins through its secretion system, thereby regulating the physiological functions of host cells to facilitate invasion and intracellular propagation. As one of the most important effector proteins secreted by *S.* Typhimurium, SopB is an inositol phosphatase that hydrolyzes a variety of phosphoinositides into 1-phosphatidyl-1D-myo-inositol 5-phosphates and orthophosphates on the host cell membrane during *S.* Typhimurium invasion (Khoo et al., 2015). SopB was reported to affect multiple cellular pathways during infection, including membrane ruffling, as well as inhibiting lysosome fusion with *Salmonella*-containing vacuoles (SCVs; Bakowski et al., 2010). Consequently, we were also interested in the function of SopB within the extracellular vesicles obtained from *S.* Typhimurium-infected Henle-407 cells. We hypothesized that SopB might facilitate the infection and the spread of *S.* Typhimurium to adjacent cells or long-distance host cells by changing their susceptibility to infection.

**Table 2:** Summary of 10 notable proteins identified in extracellular vesicles, including outer membrane structural proteins and *Salmonella* effector proteins.

**No.**	**UniProt entry**	**Gene**	**Protein name**	**Function**
**1**	H9L451	*bamB*	Outer membrane protein assembly factor BamB	Part of the outer membrane protein assembly complex, which is involved in assembly and insertion of beta-barrel proteins into the outer membrane
**2**	Q8ZN72	*nlpB*	Outer membrane protein assembly factor BamC	Same as above
**3**	Q8ZMW8	*yfiO*	Outer membrane protein assembly factor BamD	Constitutes, with BamA, the core component of the assembly machinery.
**4**	Q8ZRW0	*lptD*	LPS-assembly protein LptD	Together with LptE, is involved in the assembly of lipopolysaccharide (LPS) at the surface of the outer membrane
**5**	Q8ZQZ7	*lptE*	LPS-assembly lipoprotein LptE	Together with LptD, is involved in the assembly of lipopolysaccharide (LPS) at the surface of the outer membrane.
**6**	Q7CR87	*surA*	Chaperone SurA	Chaperone involved in the correct folding and assembly of outer membrane proteins.
**7**	Q8ZLR9	*yhbN*	Lipopolysaccharide export system protein LptA	May form a bridge between the inner membrane and the outer membrane, *via* interactions with LptC and LptD, thereby facilitating LPS transfer across the periplasm.
**8**	O30916	*sopB*	Inositol phosphate phosphatase SopB	It is one of the known effectors injected by *Salmonella* into the host cell and is required for invasion and for an efficient generation and maintenance of *Salmonella*-containing vacuole (SVC).
**9**	Q56026	*sipD*	Cell invasion protein SipD	Required for translocation of effector proteins *via* the type III secretion system SPI-1, which is essential for an efficient bacterial internalization. Probably acts by modulating the secretion of SipA, SipB, and SipC.
**10**	P35672	*sctC*	Type 3 secretion system secretin	Component of the type III secretion system (T3SS), also called injectisome, which is used to inject bacterial effector proteins into eukaryotic host cells

### Extracellular vesicles were successfully taken up by macrophages

**Figure 4:**
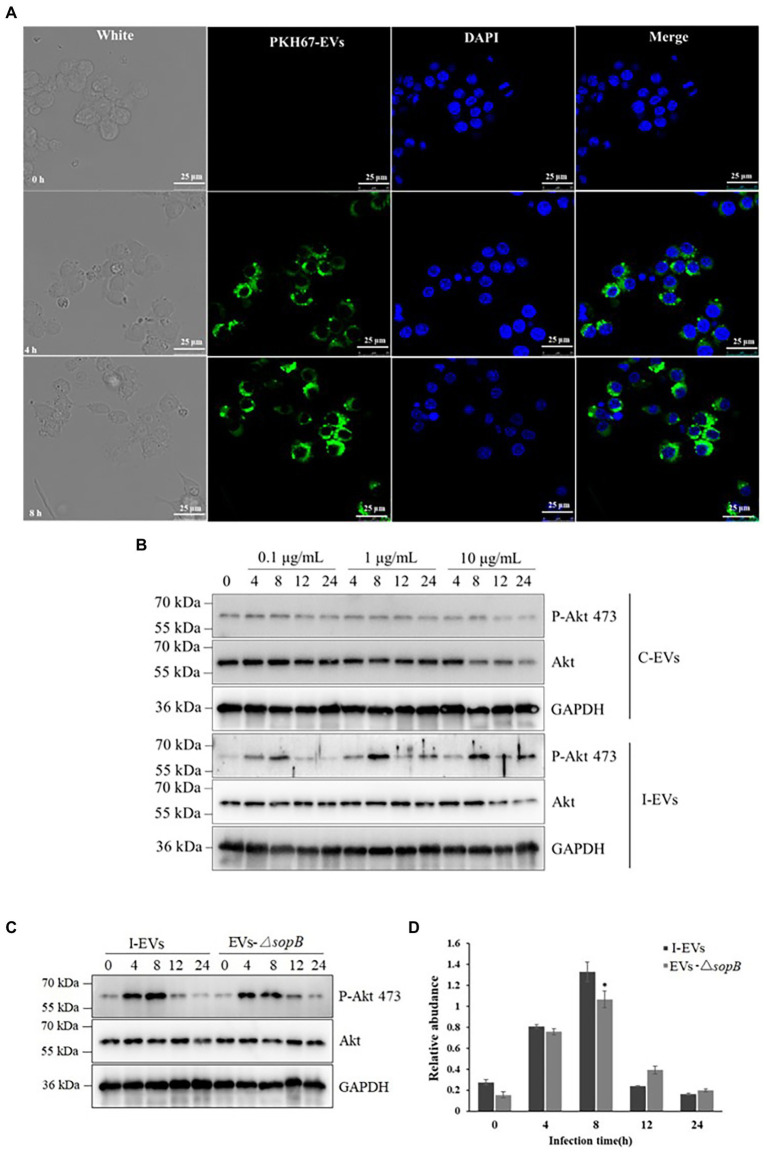
Functional verification of extracellular SopB taken up by RAW264.7 macrophages. **(A)** The RAW264.7 macrophages were incubated for 4 and 8 h (*n* = 3). DAPI and PKH67 were used to stain the macrophages and extracellular vesicles, respectively. The scale bar is 25 μm. **(B)** Phosphorylation of Akt at position 473 in RAW264.7 macrophages incubated with various concentrations of extracellular vesicles for different lengths of time. **(C)** Comparison of phosphorylation of Akt at position 473 in RAW264.7 macrophages incubated with extracellular vesicles prepared from Henle-407 cells infected with wild-type or Δ*sopB* mutant *S*. Typhimurium. **(D)** Densitometric quantification of the P-Akt 473/Akt ratio using c-Image. *P < 0.05.

Extracellular Vesicles secreted by different types of cells preferentially interact with specific target cells (Fitzner et al., 2011), and they exert their influence through two modes of action. Firstly, proteins on the surface of extracellular vesicles bind to and activate associated receptors on the membrane of the recipient cells, which do not take up extracellular vesicles (Cossetti et al., 2014; Gabrielli et al., 2015). In the second mode, extracellular vesicles can transfer their cargo into the cytoplasm of recipient cells *via* direct fusion with the plasma membrane or *via* endocytosis by recipient cells (Skokos et al., 2003). To investigate the mode of action of the extracellular vesicles produced during *Salmonella* infection, we incubated RAW 264.7 macrophages with the extracellular vesicles that were isolated from Henle-407 cells post-infection. Firstly, we stained the extracted extracellular vesicles using the lipid dye PKH67 (Wu et al., 2019), then incubated them with RAW264.7 macrophages for 4 and 8 h, and finally performed laser confocal microscopy observation after staining the macrophages using the fluorescent dye DAPI. The aggregation of extracellular vesicles (green fluorescence) around the nuclei of RAW264.7 macrophages (blue fluorescence) could be observed after extracellular vesicles were incubated with the macrophages for 4 h, and the intensity of green fluorescence obviously rose with prolonged incubation for 8 h (Figure 4A; Supplementary Figure S1). These results clearly demonstrated that macrophages could successfully take up extracellular vesicles released by *S.* Typhimurium-infected Henle-407 epithelial cells, and the numbers of the internalized extracellular vesicles increased with the incubation time. Therefore, the extracellular vesicles of *Salmonella*-infected cells appear to exert their effect through the second mode of action, i.e., by directly fusing with recipient cells.

### Extracellular SopB moderately induced Akt phosphorylation in RAW264.7 macrophages

As shown in Table 2, the proteins within extracellular vesicles released by Henle-407 epithelial cells upon *S.* Typhimurium infection included SopB. As an example of *S.* Typhimurium specific proteins within extracellular vesicles that may exert different roles after entering into recipient RAW264.7 macrophages, the role of SopB was validated. It is well known that SopB specifically induces Akt phosphorylation at position 473 during *S.* Typhimurium infection, which in turn activates Akt/mTOR signaling in the host cell to inhibit apoptosis and facilitate the proliferation of intracellular bacteria (Cooper et al., 2011). Therefore, we verified whether the extracellular vesicles containing SopB effector protein could induce Akt phosphorylation at position 473 in RAW264.7 macrophages. To test this, we incubated the RAW264.7 macrophages with 0.1, 1, or 10 mg/l of extracellular vesicles released from *S.* Typhimurium-infected Henle-407 cells for 0, 4, 8, 12, or 24 h. As a negative control, macrophages were incubated with the same amounts of extracellular vesicles acquired from uninfected Henle-407 cells. The results showed that the extracellular vesicles from the uninfected group were unable to induce Akt phosphorylation (Figure 4B), while the extracellular vesicles from infected cells induced Akt phosphorylation in RAW264.7 cells at all tested concentrations. Notably, the phosphorylation level of Akt showed a trend of first rising and then falling, with a peak at 8 h at all tested extracellular vesicle concentration. Higher phosphorylation of Akt was induced by extracellular vesicles at the dose of 1 and 10 μg/ml than at 0.1 μg/ml. However, the effect of 1 and 10 μg/ml extracellular vesicles was comparable. We therefore speculated that the amount of extracellular vesicles that could be taken up by the fixed amount of cells might be limited even if a high concentration of extracellular vesicles was used.

It has been previously established that exosomes serve as vehicles for intercellular signal transmission, and they contain a wide array of diverse molecules (Jeppesen et al., 2019). As shown in Figure 4B, although the phosphorylation of Akt at position 473 in RAW264.7 macrophages could be elicited by the extracellular vesicles of infected Henle-407 cells, this experiment did not directly confirm the role of SopB within extracellular vesicles. To further test the hypothesis that SopB is the main functional protein in the extracellular vesicles responsible for Akt phosphorylation, we compared the phosphorylation of Akt at position 473 in RAW264.7 macrophages incubated with extracellular vesicles prepared from Henle-407 cells infected with wild-type or *ΔsopB* mutant *S.* Typhimurium (Figure 4C). Consistent with Figure 4B, the level of Akt phosphorylation was activated since 4 h post co-incubation, peaked at 8 h and significantly declined after that as shown in Figure 4C. This tendency was accorded with the characteristics of P-Akt acting as an activator of signaling transduction pathway. Distinct with the equally activated in RAW264.7 cells after incubation with wild type or Δ*sopB* mutant extracellular vesicles after 4 h of incubation, it was seen that the peak of Akt phosphorylation were obviously observed by extracellular vesicles acquired from wild-type *S.* Typhimurium infected Henle-407 cells compared with those from cells infected with the Δ*sopB* mutant after 8 h of incubation. Densitometric quantification indicated that the phospho-Akt 473 was 20 percent higher in the wild type than in the Δ*sopB* group (*p* < 0.05; Figure 4D). However, this activation of Akt phosphorylation disappeared with the prolong of incubation time after 8 h. As expected, these results indicated that the *Salmonella* effector SopB contributed to the activation of Akt phosphorylation when the *S*. Typhimurium infection acquired extracellular vesicles co-incubated with RAW264.7 macrophages in comparison with Δ*sopB* group, due to the reason that SopB is the only known effector protein of *S.* Typhimurium that induces Akt phosphorylation (Cooper et al., 2011). Based on it, we could conclude that besides SopB, there also existed other unknown components within extracellular vesicles also engaged in the activation of Akt phosphorylation which might be derived from Henle-407 cells during the interaction between pathogenic bacteria and host cells owing to the equally activated Akt phorylation at 4 h post extracellular vesicles incubation (Figures 4C,D). In summary, our results distinctly indicated the role of SopB in Akt phosphorylation during *S*. Typhimurium infection that delivered by extracellular vesicles secretion pathway.

## Discussion

### *Salmonella* infection modulated the composition of proteins within extracellular vesicles of host cells

Extracellular Vesicles are secreted vesicles with diameters ranging from 30 to 200 nm. They are derived from the endosomal membrane system and have important roles in cellular biological processes (Pegtel and Gould, 2019). Previous studies of the extracellular proteome of human macrophages infected with *S.* Typhimurium indicated that some of the proteins secreted by macrophages upon *S.* Typhimurium infection are released *via* exosomes, which trigger proinflammatory effects in the early phase of infection (Bhatnagar et al., 2007; Hui et al., 2018). This stimulation of proinflammatory effects by exosomes was thought to be partially attributed to the encapsulated lipopolysaccharide within extracellular vesicles (Ria et al., 2009). In addition, it was believed that extracellular vesicles could activate innate and acquired immune responses due to the presence of multiple components of the bacterial cells of *Salmonella*. However, few systematic analysis of bacterial components contained within extracellular vesicles secreted by host cells upon *Salmonella* infection has been performed in previous studies except a recent research about host extracellular vesicles derived from *Salmonella*-infected cells stimulate pathogen-specific responses (Hui et al., 2021). Until now, the role of extracellular vesicles in immune responses against bacterial pathogens were mainly analyzed in both Hui and our research. But differently, in Hui’s research, extracellular vesicles were secreted by *Salmonella*-infected macrophages, while in our research, extracellular vesicles were isolated from *Salmonella*-infected epithelial cells. It is well known that human intestinal epithelium is the first target of *Salmonella* invasion and colonization after ingestion of contaminated food or water (Jones et al., 2008; Hendriksen et al., 2011; Kulkarni Devesha et al., 2018). However, macrophage is indeed executor responsible for cell defense by immune systems, which implicates that the above two studies are both important and complementary in understanding how the host responding to *S.* Typhimurium infection by means of extracellular vesicle’s pathway within various types of host cells. Hui et al. identified 373 and 325 species of *Salmonella* antigens at 24 hpi and 48 hpi respectively, while our results showed that 409 *Salmonella* proteins (Supplementary Table S2) were identified within the extracellular vesicles secreted by *Salmonella* infected Henle-407 cells at 8 hpi. In our opinion, this distinction between the number of proteins was normal based on the different types of cell lines which exert distinct functions during *S*. Typhimurium infection and the time of infection was different meanwhile, which might also be a reason for this distinction. Besides, we found that some of the *Salmonella* proteins analyzed by Hui *et al*, were similar to ours results, such as SopB, SlyB, nlpB, OmpA, OmpC, IroN, FepA, tolC, tolB, traT, yajG *et al* (Supplementary Table S2; Table 2), from which we could conclude that some of the *Salmonella* proteins from extracellular vesicles should be general between different types *Salmonella*-infected cells, strengthening our conclusion that label free relative quantitative proteomics reveals extracellular vesicles as a vehicle for *Salmonella* effector protein delivery. Furthermore, we focused on the role of extracellular vesicles in effector protein delivery while Hui et al. found the extracellular vesicles could induce Th1-type immunity to Gram-negative bacteria in mice, which provides wider insights for our further research on the role of extracellular vesicles.

In our present study, extracellular vesicles were successfully isolated from cultured human epithelial cells by a combination of two classical methods (Figure 1), which laid a solid foundation to assure the purity of isolated extracellular vesicles, which was a key factor for further proteomic analysis (Chen et al., 2017). After extracellular vesicle isolation, label-free relative quantitative proteomic analysis was used to identify the bacterial proteins present in extracellular vesicles released from *Salmonella*-infected Henle-407 cells. A total of 409 *Salmonella*-specific proteins were identified, which were specifically present in extracellular vesicles released by the host cells after *Salmonella* infection.

Why would such a high number of specific bacterial proteins enter into host extracellular vesicles during *Salmonella* infection? Recent studies have deduced two major mechanisms by which proteins are targeted to the intraluminal vesicles of MVBs for subsequent release as part of exosomes. One involves ubiquitination of proteins which is recognized by the ESCRT (endosomal sorting complex required for transport) machinery for subsequent trafficking and retention of proteins in the intraluminal vesicles (Simons and Raposo, 2009). The other mechanism involves aggregation of membrane proteins to form microdomains, which may stabilize the exosomal membrane domains (Simons and Raposo, 2009). It was reported that *Salmonella* can modulate the ubiquitination pathway of host cells by effector proteins secreted through the T3SS, such as E3 ligases SopA, SspH1, SspH2 and SlrP (Ria et al., 2009), as well as the deubiquitinase SseL (Rytkoenen et al., 2007). Furthermore, the T3SS effectors SopA, SopE, and SopB/SigD are all translocated and can work together with ubiquitin to interfere with the immune responses of host cells (Wang et al., 2018). Here, we identified the *Salmonella* proteins SopA, SopB and SopE in extracellular vesicles from the infected group (although SopA and SopE appeared only once in three replicates). We speculate that these proteins may aggregate within the phagosome by inducing host cell ubiquitination pathways and auto-ubiquitination, leading to their trafficking into extracellular vesicles *via* the endocytic network. In addition to these known pathways, there is evidence of possible ESCRT-independent transport mechanisms (Trajkovic et al., 2008), but whether some or all of the *Salmonella* proteins use these alternative targeting pathways requires further studies in the future.

Since a number of *Salmonella* proteins within extracellular vesicles were released from *Salmonella*-infected human intestinal epithelial cells, we analyzed the GO and KEGG pathways of these *Salmonella*-specific proteins to explore the main direction of their functions. Interestingly, the molecular functions of these secretory proteins and the number of enriched proteins were basically consistent with the analysis results of all proteins (Figures 2D, 3B). This suggested that there might be effector proteins actively secreted by *Salmonella* that play major regulatory roles among the proteins in extracellular vesicles released by infected host cells. Previous studies have shown that *Salmonella* effector proteins can affect the host immune system and can function to either promote an effective immune response or potentially inhibit aspects of this response (Herhaus and Dikic, 2018). However, few studies defined the effector protein components present in exosomes post infection by *Salmonella*. To what degree extracellular vesicles promote or inhibit immunity would likely depend on what *Salmonella* effector proteins are present in the exosomes at any given time. Notably, we identified 10 putative effector proteins from 75 actively secreted proteins of *Salmonella*, including SopB, SipD, and SctC, as well as 7 proteins involved in bacterial outer membrane assembly (Table 2). Based on this, we speculated that the release of extracellular vesicles by host cells after *Salmonella* infection might be used to transfer effector proteins to neighboring cells and may be associated with the spread of *Salmonella* infection and further intracellular replication.

### Extracellular vesicles mediate the induction of Akt phosphorylation in neighbor cells by SopB during *Salmonella* infection

*Salmonella* normally secretes the effector protein SopB through T3SS, which activates the host cell kinase Akt/mTOR signaling pathway during bacterial invasion, thereby inhibiting apoptosis of intestinal epithelial cells and providing an opportunity for intracellular *Salmonella* survival (Owen et al., 2014). In addition, SopB plays multiple functions including the activation of SH3-containing guanine nucleotide exchange factor (SGEF) on the recipient cell membrane to drive activation of the exchange factor RhoG, which in turn drives actin-induced cytoskeletal reorganization of the recipient cell, thereby allowing *Salmonella* to be internalized into the recipient cell (Galán, 2006). It was reported that SopB is recruited to the plasma membrane *via* ubiquitination catalyzed by the host ubiquitin ligase TRAF6 (Ruan et al., 2014, 2016). Here, we found a novel mode through which SopB may exert its function, since it is transferred into extracellular vesicles after *Salmonella* infects epithelial cells. When cultured macrophages were incubated with extracellular vesicles isolated from the infected group, there was increased phosphorylation of Akt protein in a time- and dose-dependent manner. In addition, the experimental results also showed that the presence of other proteins in addition to SopB within the extracellular vesicles of the infected group could also cause the phosphorylation of Akt protein, even in extracellular vesicles derived from cells infected with a *ΔsopB* knockout mutant, which is in agreement with the view that exosomes contain many bacterial and host-derived proteins. Therefore, we inferred that small extracellular vesicles (exosomes) maybe a previously overlooked route for the transfer of SopB from infected cells to macrophages or neighbor cells during *Salmonella* infection.

## Conclusion

The label-free quantitative proteomic analysis of extracellular vesicles released from infected epithelial cells as described in this study was the first venture into dissecting their protein composition, and validating extracellular vesicles as a way for *Salmonella* to deliver virulence proteins to facilitate infection. Future studies may focus on defining how extracellular vesicle composition changes over time, especially during *in vivo* infection, as well as its relationship with the bacterial burden and the stage of infection, aiming to better understand the roles of extracellular vesicles during the course of *Salmonella* infection.

## Data availability statement

The datasets presented in this study can be found in online repositories. The mass spectrometry proteomic data have been deposited with the ProteomeXchange Consortium (http://proteomecentral.proteomexchange.org) via the iProX partner repository (Ma et al. 2019) with the dataset identifier PXD032776.

## Author contributions

HR and BZ conceived and designed the experiments. TW and BZ drafted the manuscript. BZ and JL collected the samples. BZ, JL and HW performed the experiments. TW, BZ, JL, and AH collected and analyzed the data. JQ and HR participated in study design, technical guidance, and coordination. All authors contributed to the article and approved the submitted version.

## Funding

This work was supported by the General Program of the National Natural Science Foundation of China (Grant number: 31870122), the Youth Program of the National Natural Science Foundation of China (Grant number: 21908168), and the Natural Science Foundation of Tianjin (Grant number: 18JCYBJC96000).

## Conflict of interest

The authors declare that the research was conducted in the absence of any commercial or financial relationships that could be construed as a potential conflict of interest.

## Publisher’s note

All claims expressed in this article are solely those of the authors and do not necessarily represent those of their affiliated organizations, or those of the publisher, the editors and the reviewers. Any product that may be evaluated in this article, or claim that may be made by its manufacturer, is not guaranteed or endorsed by the publisher.

## Supplementary material

The Supplementary material for this article can be found online at: https://www.frontiersin.org/articles/10.3389/fmicb.2022.1042111/full#supplementary-material

## Footnotes

^1^ http://www.blast2go.de
