# Supplementary material for: Label-free relative quantitative proteomics reveals extracellular vesicles as a vehicle for Salmonella effector protein delivery
Source: Front Microbiol. 2022 Dec 15;13:1042111. doi: 10.3389/fmicb.2022.1042111 (PMC9797957; doi:10.3389/fmicb.2022.1042111)
Supplement: Supplementary file 1 [file Data_Sheet_1.ZIP › Supplementary materials/Figure S1.docx]

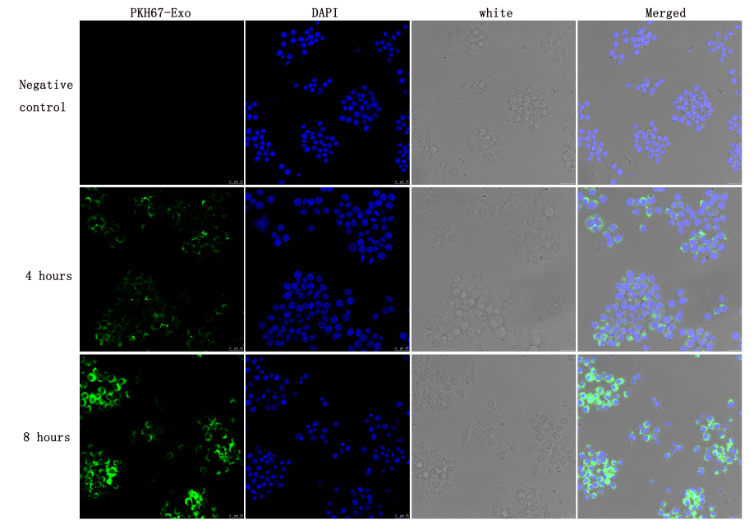


Figure S1. The RAW264.7 macrophages were incubated for 4 and 8 h (n = 3). DAPI and PKH67 were used to stain the macrophages and exosomes, respectively. The scale bar is 25μm. The magnification times is 400.
