# Supplementary material for: Label-free relative quantitative proteomics reveals extracellular vesicles as a vehicle for Salmonella effector protein delivery
Source: Front Microbiol. 2022 Dec 15;13:1042111. doi: 10.3389/fmicb.2022.1042111 (PMC9797957; doi:10.3389/fmicb.2022.1042111)
Supplement: Supplementary file 1 [file Data_Sheet_1.ZIP › Supplementary materials/Table S3-S4.docx]

**Table S3** Classical secretory protein

| **UniProt ID** | **Protein Name** | **Gene Name** | **SignalP** | **SecretomeP** |
| --- | --- | --- | --- | --- |
| P0A2J9 | Trk system potassium uptake protein TrkA | trkA | YES | NO |
| P43669 | Tail-specific protease | Prc | YES | NO |
| Q7CQU5 | Thioredoxin reductase | TrxB | YES | NO |
| Q7CR87 | Chaperone SurA | SurA | YES | NO |
| Q8ZK66 | UDP-N-acetylmuramate--L-alanyl-gamma-D-glutamyl-meso-2,6-diaminoheptandioate ligase | Mpl | YES | NO |
| Q8ZM72 | Proline aminopeptidase P II | PepP | YES | NO |
| Q8ZNG7 | Periplasmic glycerophosphodiester phosphodiesterase | GlpQ | YES | NO |
| Q8ZP17 | D-amino acid dehydrogenase | DadA | YES | NO |
| A0A0F6B6W5 | Outer membrane channel protein | TolC | YES | YES |
| H9L419 | Putative periplasmic binding transport protein | FliY | YES | YES |
| H9L451 | Outer membrane protein assembly factor BamB | BamB | YES | YES |
| P06202 | Periplasmic oligopeptide-binding protein | OppA | YES | YES |
| P0A1X0 | Outer membrane lipoprotein SlyB | SlyB | YES | YES |
| P0A231 | Phospholipase A1 | PldA | YES | YES |
| P0A2C5 | Ribose import binding protein RbsB | RbsB | YES | YES |
| P26265 | 2,3-cyclic-nucleotide 2-phosphodiesterase/3-nucleotidase | CpdB | YES | YES |
| P26478 | Maltose operon periplasmic protein | MalM | YES | YES |
| P30752 | Outer-membrane lipoprotein LolB | LolB | YES | YES |
| P35672 | Type 3 secretion system secretin | SctC | YES | YES |
| P37409 | Vitamin B12 transporter BtuB | BtuB | YES | YES |
| P40827 | Murein hydrolase activator NlpD | NlpD | YES | YES |
| P67557 | Glucans biosynthesis protein G | MdoG | YES | YES |
| Q56078 | Periplasmic beta-glucosidase | BglX | YES | YES |
| Q7CP68 | Hyperosmotically inducible periplasmic protein | OsmY | YES | YES |
| Q7CPJ3 | Putative inner membrane lipoprotein | STM3580 | YES | YES |
| Q7CPT7 | Periplasmic L-asparaginase II | AnsB | YES | YES |
| Q7CQ32 | Lipoprotein | VacJ | YES | YES |
| Q7CQE1 | Putative outer membrane protein | Slp | YES | YES |
| Q7CQQ5 | Putative outer membrane lipoprotein | STM1254 | YES | YES |
| Q7CQV7 | Putative periplasmic protein | YbiS | YES | YES |
| Q7CQW9 | Peptidoglycan-associated protein | Pal | YES | YES |
| Q8ZKX5 | Phosphate-binding protein PstS | PstS | YES | YES |
| Q8ZLA9 | Dipeptide transport protein | DppA | YES | YES |
| Q8ZLL6 | Peptidyl-prolyl cis-trans isomerase | FkpA | YES | YES |
| Q8ZLR9 | Lipopolysaccharide export system protein LptA | YhbN | YES | YES |
| Q8ZMN0 | TonB-dependent siderophore receptor protein | IroN | YES | YES |
| Q8ZMW8 | Outer membrane protein assembly factor BamD | YfiO | YES | YES |
| Q8ZN72 | Outer membrane protein assembly factor BamC | NlpB | YES | YES |
| Q8ZNH6 | Periplasmic nitrate reductase | NapA | YES | YES |
| Q8ZP50 | Outer membrane protein W | OmpW | YES | YES |
| Q8ZPT3 | Putative outer membrane protein | STM1328 | YES | YES |
| Q8ZQ08 | Penicillin-binding protein activator LpoB | LpoB | YES | YES |
| Q8ZQD3 | Anaerobic dimethyl sulfoxide reductase, subunit A | DmsA | YES | YES |
| Q8ZQT5 | Tol-Pal system protein TolB | TolB | YES | YES |
| Q8ZQZ7 | LPS-assembly lipoprotein LptE | LptE | YES | YES |
| Q8ZR40 | Outer membrane porin, receptor for ferric enterobactin (Enterochelin) and colicins B and D | FepA | YES | YES |
| Q8ZRA6 | Acridine efflux pump | AcrA | YES | YES |
| Q8ZRW0 | LPS-assembly protein LptD | LptD | YES | YES |
| Q93GL9 | TraT complement resistance protein | TraT | YES | YES |
| Q9ZF60 | Glutamate/aspartate import solute-binding protein | GltI | YES | YES |
| A0A0F6AXS1 | Chaperone SurA | YajG | YES | YES |

**Table S4** Nonclassical secretory protein

| **UniProt ID** | **Protein Name** | **Gene Name** | **SignalP** | **SecretomeP** |
| --- | --- | --- | --- | --- |
| A0A0F6B7M3 | 30S ribosomal protein S14 | rpsN | NO | YES |
| O30916 | Inositol phosphate phosphatase SopB | sopB | NO | YES |
| P0A1R0 | RNA-binding protein Hfq | Hfq | NO | YES |
| P0A2C7 | Spermidine/putrescine-binding periplasmic protein | potD | NO | YES |
| P0A2F4 | Superoxide dismutase [Fe] | sodB | NO | YES |
| P0A2F6 | Single-stranded DNA-binding protein 1 | Ssb | NO | YES |
| P0A2I1 | DNA topoisomerase 1 | topA | NO | YES |
| P0A9Y9 | Cold shock-like protein CspC | cspC | NO | YES |
| P0AA07 | Phosphocarrier protein HPr | ptsH | NO | YES |
| P15888 | Oxygen-insensitive NAD(P)H nitroreductase | nfsB | NO | YES |
| P17750 | Catalase-peroxidase | katG | NO | YES |
| P43019 | Superoxide dismutase [Mn] | soda | NO | YES |
| P66491 | 30S ribosomal protein S19 | rpsS | NO | YES |
| Q56026 | Cell invasion protein SipD | sipD | NO | YES |
| Q7CQ23 | Thioredoxin dependent thiol peroxidase | Bcp | NO | YES |
| Q8XFG8 | Peptidyl-prolyl cis-trans isomerase | ppiB | NO | YES |
| Q8ZKE2 | Fumarate hydratase class I | fumB | NO | YES |
| Q8ZKY4 | Membrane protein insertase YidC | yidC | NO | YES |
| Q8ZLP3 | Cell shape-determining protein MreC | mreC | NO | YES |
| Q8ZN53 | Cytoskeleton protein RodZ | rodZ | NO | YES |
| Q8ZP37 | Nitrate reductase 1, alpha subunit | narG | NO | YES |
| Q8ZPL8 | Putative periplasmic protein | ydgA | NO | YES |
| Q8ZQS1 | Aldose 1-epimerase | galM | NO | YES |
| Q8ZRQ3 | Penicillin-binding protein 1B | mrcB | NO | YES |
